# Patient Engagement in Provincial and Territorial Medical Association Decision-Making: A Rapid Scoping Review

**DOI:** 10.7759/cureus.54400

**Published:** 2024-02-18

**Authors:** Ryan Brown, Samantha Graham, Jennifer Girard, Noelle Moulaison

**Affiliations:** 1 Emergency Medicine, Dalhousie University, Halifax, CAN; 2 Health System Integration and Practice Support, Doctors Nova Scotia, Dartmouth, CAN; 3 Policy and Physician Wellness, Doctors Nova Scotia, Dartmouth, CAN

**Keywords:** patient-centered care, advocacy, physician compensation, patient partners, medical society, medical association

## Abstract

In recent years health systems have engaged patient partners in decision-making. Provincial and Territorial Medical Associations (PTMAs) are the sole bargaining agents for physicians. PTMA negotiations with governments are often seen as insular. Adding the patient perspective could add tremendous value to negotiating committees, etc. as PTMAs look to advocate for person-centered care provided by their members. Using rapid scoping review methodology, PubMed was searched for studies reporting on the use of patient partners in PTMA decision-making. Title and abstract screening were conducted by a single reviewer with full-text review screened by two reviewers.

The search yielded 231 titles with 10 moving to full-text review and ultimately no titles meeting inclusion criteria. This empty scoping review has identified a paucity of literature reporting on patient engagement in PTMA decision-making. Further research is required to determine the utility of introducing patient partners in this capacity.

## Introduction and background

The integration of patient partners in health systems marks a significant shift from a paradigm of paternalism to one that values patient autonomy and involvement in decision-making [1]. Engaging patient partners in clinical decision-making and service delivery design has been shown to improve outcomes and satisfaction [2].

Provincial and Territorial Medical Associations (PTMAs) are the sole bargaining agents for physicians with the various Jurisdictional Departments of Health in Canada and are the collective voices of physicians from an advocacy standpoint. Physicians are predominantly independent contractors and negotiations on compensation between PTMAs and governments are often seen as inward-looking and perceived as not system-minded.

Physicians, by virtue of their education and level of responsibility, are well compensated in Canada. This often leads to less sympathy from the public for less-than-favorable negotiated increases for physicians than for lower-earning health professionals with equally unfavorable increases [3-5]. Physicians spend their early adulthood forgoing years of earnings and accruing high debt loads and are subject to inflationary pressures in their personal and professional lives. These facts are often lost on the government and the public. Implementing patient partners, trained in physician compensation, as consultants to PTMA boards, negotiation teams, and committees would provide a non-physician voice to both government and the public on these issues.

Conversely, as siloed as matters of physician remuneration and contract negotiations can be from the general public, the insular nature of these proceedings involving physicians and PTMA staff has created a risk of bias. Adding the patient perspective and a view from the outside looking in could add tremendous value to negotiating committees, etc. as PTMAs look to advocate for person-centered care provided by their members, doing what is best for the system at large.

Our objective is to describe to what extent PTMAs have engaged patient partners in decision-making. The scoping review methodology was chosen as appropriate to gauge the extent and range of literature on a topic and to identify gaps in the existing scientific and grey literature [6,7].

## Review

Methods

Protocol and Registration

A preliminary search of MEDLINE, the Cochrane Database of Systematic Reviews, and Joanna Briggs Institute (JBI) Evidence Synthesis was conducted and no current or underway systematic reviews or scoping reviews on the topic were identified.

The review was conducted in accordance with the JBI methodology for scoping reviews and guided by The Cochrane Handbook for Accelerated Reviews, the JBI Manual for Synthesizing Evidence, and the Preferred Reporting Items for Systematic Reviews and Meta-Analyses Extension for Scoping Reviews (PRISMA-ScR) checklist for reporting [8-10].

The review protocol was registered on the Open Science Framework platform (registration number: 10.17605/OSF.IO/PWBZ6).

Eligibility Criteria

This review explores the concept of engaging patients in PTMA decision-making. The role of the patient partner may include sitting on PTMA boards, standing and ad hoc committees, negotiating teams, or as stand-alone resources. Any review in the title and abstract screening phase that made reference to a medical association or analogous organization engaging with patients moved on to full-text review. Data abstraction for full-text review focused specifically on engaging patients in PTMA decision-making.

Types of Sources

This scoping review considered both quantitative and qualitative reports. Systematic reviews that met the inclusion criteria were eligible, if aligned with the research question, as were narrative reviews. Text and opinion papers were also considered for inclusion in this scoping review. This review did not consider grey literature or conference proceedings.

Restrictions

We set limits for studies published in English since the year 2000 as per the expedited nature of this review. Rapid scoping review methods were utilized as the authors had a baseline sense that there would be little published literature in this area and justified putting resources toward a rapid scoping review in lieu of a full scoping review.

Information Sources

For efficiency, and as per rapid scoping review methodology, this review utilized PubMed as the primary platform to search for pertinent literature. Furthermore, PubMed is a large medical literature database that has been shown to have broad overlap with other healthcare journal databases [11-13]. As previously stated, an a priori preliminary search of the Cochrane Database of Systematic Reviews and JBI Evidence Synthesis was conducted to identify current or underway systematic reviews or scoping reviews on the topic.

Search

The following search strategy was developed by an experienced evidence synthesis researcher and run via PubMed on December 6, 2023: (patient(tiab) OR stakeholder(tiab) OR caregiver(tiab) OR carer(tiab) OR client(tiab) OR person(tiab)) AND (engagement(tiab) OR partner(tiab) OR advocate(tiab) OR surrogate(tiab) OR collaborate(tiab)) AND (medical society(tiab) OR medical association(tiab) OR medical college(tiab)).

The filters are set to English and cover the period from January 1, 2000, to December 1, 2023.

Selection of Evidence

Studies moved from title and abstract screening to full-text only if there was any mention of a medical association or analogous organization engaging with patients. Titles were to be included for analysis only if the article specifically commented on engaging patients in PTMA decision-making.

Results

**Figure 1:**
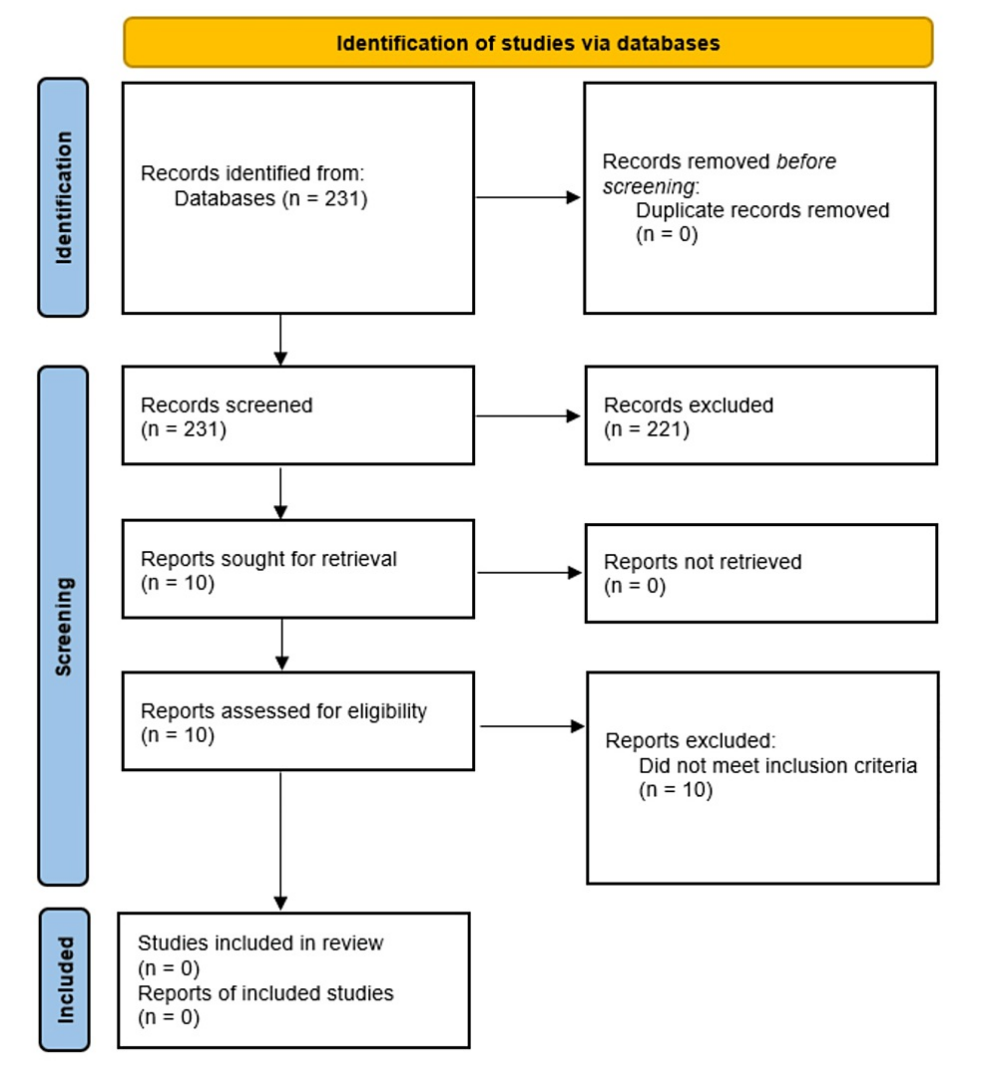
PRISMA flow diagram depicting the selection of studies PRISMA: Preferred Reporting Items for Systematic Reviews and Meta-Analysis

The search of PubMed yielded 231 titles, with no duplicates found. Ten studies moved on to full-text review, with none reaching the eligibility criteria for analysis. Refer to Figure 1 for the PRISMA flow diagram of results [14]. This rapid scoping review exploring patient engagement in PTMA decision-making can be designated as an “empty” review.

Discussion

Patient partnerships in healthcare and medicine are becoming commonplace, shifting from a rigid, siloed approach to a more collaborative framework. This approach acknowledges patients not merely as passive recipients of care but as active, informed participants in their healthcare journey. From a health policy standpoint, integrating patient partnerships holds profound implications, fostering a more patient-centered healthcare system and enhancing the overall quality of care [1,15-17].

At their core, patient partnerships advocate for shared decision-making, where patients are empowered to contribute to treatment plans and care strategies [18]. By engaging patients in discussions about their preferences, values, and goals, healthcare providers can tailor interventions to align with individual needs. This collaborative dynamic not only enhances treatment adherence but also cultivates a sense of ownership and accountability, leading to improved health outcomes [19].

From a policy perspective, promoting patient partnerships demands systemic changes that recognize and prioritize patient engagement. Policies should advocate for the integration of patient perspectives in healthcare planning, research, and service delivery [1,19]. This involves cultivating a healthcare ecosystem that values patient input, ensuring that institutional structures facilitate and support active patient involvement [1].

Moreover, health policies need to emphasize education and training for healthcare professionals and organizations to effectively engage in patient partnerships. Training programs should focus on communication skills, cultural competence, and shared decision-making techniques to enable healthcare providers and organizations to effectively collaborate with patients from diverse backgrounds and with varying health literacy levels [1,20].

Policy frameworks must also address barriers to patient engagement, including systemic inequalities, accessibility issues, and disparities in healthcare delivery [19]. Policies should strive to mitigate these barriers by promoting inclusive practices, ensuring equitable access to information, and providing resources that support patient involvement regardless of socioeconomic status or geographic location [19,21].

Patient engagement has been shown to have tangible benefits. Laurance et al. [22] outlined patient partner integration case studies from diverse clinical settings (mental health, genetic screening, primary maternity care, and acute care) all demonstrating reduced costs, improved clinical outcomes, and enhanced access. In Canada, the Canadian Institutes of Health Research (CIHR) has made patient partner engagement a priority via its Patient Engagement Framework [23]. Patient engagement early in the research process has been shown to add transparency, lead to improved outcomes, and guide research questions in new directions [23-25]. When embedded early on in research teams patient partners often feel their contribution to lived experience is valued and their input improves the research process [25].

Conversely, there are instances documented when patient partnerships are not as fruitful. While this may lead to less-than-desirable outcomes of a patient engagement strategy, these outcomes are typically due to issues with the strategy itself [26]. Lack of appropriate training for both the patient partners and staff they are to interact with is often the barriers that lead to failed projects. It must be recognized that patient partners are often vulnerable and their ideas may not heard if they do not have frontline champions to support their inclusion [26,27]. It should be noted that, overall, patient partnerships described in the literature are quite positive. While publication bias could play a part, the benefits health organizations see from engaging patient partners are not in dispute.

Our search yielded 231 titles with the majority being focused on direct clinical partnerships with doctors and in some cases nurses. A minority of articles dealt with patient engagement and partnerships in specific programs of care or healthcare organizations. The focus of this review was not clinical and the partnerships with organizations pertaining to programs of care and service delivery were narrowly focused and did not involve PTMAs. Thus, none of the results yielded by the search met the inclusion criteria for analysis. This was not surprising to the authors and this empty review demonstrates a significant gap in the knowledge pertaining to the engagement of patient partners in PTMA decision-making.

Empty systematic reviews are characterized by a lack of eligible studies or insufficient evidence to draw meaningful conclusions [28,29]. These reviews, despite rigorous methodological approaches, identify the absence of data on the topic under investigation. Understanding the implications of such reviews is key to informing research practices, policy decisions, and healthcare resource allocation [30]. Empty reviews uncover gaps in knowledge and evidence, flagging areas where further research is required. Identifying these gaps provides valuable insights for researchers and policymakers, guiding future research and funding opportunities [30,31].

In the case of this study, we did not conduct a systematic review, but a rapid scoping review. The intent of scoping reviews, as per Arksey and O’Malley’s [6] seminal work, is to scan the landscape, inform research, and identify gaps. While the notion of empty systematic reviews can be controversial concerning their scientific utility, there is a paucity of discourse on empty scoping reviews [31]. The authors would posit that, at their core, whether a scoping review yields evidence that meets inclusion for analysis, or not, the review has achieved its objective.

At the inception of this project a preliminary, unstructured grey literature search produced no evidence as to any patient partnership programs in existence within any Canadian PTMAs. It was discovered that the Canadian Medical Association (CMA), the national medical association and collective voice of physicians in Canada, does have a patient partner program branded “The Patient Voice,” as well as a patient engagement framework [32]. The CMA defines patient-partnered care as: “an authentic collaboration between decision makers; patients, healthcare providers, and informal caregivers, built upon four foundational team-based pillars including collaborative leadership, communication, situation monitoring, and shared decision making and mutual support. This collaboration must embody an equity-informed approach that ensures that all barriers that stand in the way of patient and informal caregiver engagement are addressed in a respectful manner. These pillars support a team-based culture that is important for patient-partnered individual health decision-making, designing or redesigning care delivery systems and research” [33].

The CMA acknowledges the importance of patient perspectives and emphasizes the inclusion of patients as active participants in healthcare decision-making processes. Furthermore, their Patient Engagement Framework has four components across the continuum of engagement: inform, consult, co-create, and enable [34]. This framework engages patient partners at all phases of advocacy and policy development. Unlike the PTMA model, the national association does not deal directly with physician compensation models, but this framework has the potential to be adopted by PTMAs for both their advocacy and policy-setting functions as well as their role as bargaining agents.

One such advocacy initiative this framework could be applied to at the PTMA level is a model for primary care which has been gaining support across the country since the early 2000s, the patient-centered medical home (PCMH). Often referred to as “health homes” or “health neighborhoods,” PCMHs are an approach to providing comprehensive medical care where patients have a strong connection with their physician but also have access to other allied healthcare providers who may become the most appropriate provider for their needs at any time [35].

In Nova Scotia (NS), for example, there are approaching 100 collaborative family practice teams which could include family physicians, registered nurses, social workers, dieticians, etc. [36]. These practice teams work within a "health home" model which aligns with the PCMH vision of providing comprehensive primary care [36]. While health homes have gained broad support in NS from allied health provider associations, colleges, and the provincial government, there remains an obvious voice absent from the table when discussions are had concerning logistics. Patient partner engagement in discussions about health homes and PCMHs should be explored. If the patient is to be at the center of this comprehensive model of primary care, their voice should be present in decision-making.

Limitations

Rapid review methodology in the context of scoping reviews does have inherent limitations. The review was conducted via a search of a single database and the reality exists that some evidence could have been missed which would have been eligible for analysis. PubMed was chosen for its robust nature in the context of healthcare and medicine, including policy papers, however running the search in other databases such as Embase could have improved the reach of the review. Our search was developed by a researcher with evidence synthesis and critical appraisal expertise, however, it was not vetted by a health sciences librarian. The search strategy also did not undergo peer review. These limitations were imposed by the decision to use rapid review methods due to workload and available resources.

## Conclusions

This rapid scoping review of the literature was conducted with the objective to describe to what extent PTMAs have engaged patient partners in decision-making. Our search yielded no evidence that met the inclusion criteria, thus producing an empty review. Most literature identified in this space was specific to patient partners engaging in direct clinical care, programs of care, or healthcare organizations broadly. No articles were identified discussing patient partner engagement in PTMA decision-making or matters of physician compensation; however, there is precedence set with the national association in the policy space. Although empty reviews can be contentious, in the spirit of scoping reviews being a mechanism to map the literature on a given subject, this review has met its objectives. Further research is required in this space. Given the success of patient partners in other areas of healthcare, policymakers should consider the use of patient partners in PTMA decision-making as the role has been found to have benefits in both clinical care and service delivery design.
